# Some Clinical Aspects of Granular Kidney

**Published:** 1899-03-18

**Authors:** Samuel West


### Hospital Clinics and Medical Progress.

### SOME CLINICAL ASPECTS OF GRANULAR KIDNEY.

Abstract of Lettsomian Lectures delivered before the Medical Society of London by Samuel West, M.D., F.R.C.P.

### Lecture III.

Again it has to be insisted on that granular kidney may exist for a long time without appearing to affect the health or producing symptoms, and that when the latter appear, no matter what their nature, the disease is far advanced and in its later stages. The symptoms which may be met with are multifarious, and unless there be intercurrent nephritis they may in no way suggest the presence of renal disease. They fall into two categories—the cardio-vascular and the toxæmic, and the former generally occur the first. If the disease were to last long enough the toxæmic symptoms would always occur, for they depend upon the wasting of the kidney and advance *pari passu* with it. The cardio-vascular symptoms are more or less accidental, but by their intervention death is often caused earlier than it would have been by the wasting of the kidney alone.

### Cardiac Symptoms.

The cardiac symptoms are those of heart failure; the vascular arise from hæmorrhage and its results and from the occurrence of chronic degenerative affections, especially in the nervous system, resulting from imperfect nutrition. Heart failure, showing itself by a little shortness of breath and palpitation on exertion, may be the first thing to cause anxiety. With it there may be some cardiac pain and swelling of the feet, and the pain, although usually slight, may be severe enough to be called angina. The heart may be found dilated or hypertrophied without obvious cause in the heart itself. In elderly people, of course, such symptoms may be due to atheroma, but in middle life and in the young granular kidney is their most likely cause. Heart symptoms, however, may be due, as in the case of pericarditis—a very serious complication—to conditions arising rather from the toxæmic effects of granular kidney than from its direct effect upon the cardiac mechanism.

### Vascular Symptoms.

*Aneurism* may occur in cases of granular kidney. This is a new point, but the connection is well shown by the statistics of St. Bartholomew's Hospital, where 6 per cent. of the deaths from granular kidney were found to be due to rupture of thoracic aneurisms.

*Hæmorrhage* may occur in many parts, but it produces its most serious effects when it takes place in the brain, where it may plough up the central nervous tissue to such an extent as to cause death from apoplexy, or by pressing on or destroying smaller areas may produce various forms of paralysis.

In considering the diagnostic meaning of cerebral hæmorrhage due allowance must be made in elderly persons for atheroma; but even elderly persons may have granular kidney and a little atheroma, while in middle life granular kidney is the usual cause, and, in the young, it may explain cases of cerebral hæmorrhage which would otherwise present great difficulty.

It is worth remembering that epistaxis is a common form of hæmorrhage in granular kidney, and that its diagnostic importance is often missed.

*Hæmaturia* is another important form of hæmorrhage in granular kidney. The blood is intimately mixed with the urine and is bright in colour, and probably has its common origin in the bladder. It may be so red and bright as to look almost like pure blood, but even then there are hardly ever any clots. It is not often copious, but it is common and recurrent. It has led to frequent mistakes in diagnosis, as, for example, to a diagnosis of a calculus in the bladder. Hæmorrhage may, among other places, arise from the bowels or the uterus, and in the last stages of granular kidney the patient may pass into an almost hæmophylic condition, in which slight, though continuous and almost uncontrollable, oozing takes place from various parts of the body, or from any wound or scratch in the skin.

Although the degenerations which occur in various organs in connection with granular kidney may be the direct result of innutrition from arterial disease, it must not be forgotten that in many cases they may also be of toxæmic origin.

### Chronic Renal Toxæmia.

The toxæmic symptoms of granular kidney fall into two very distinct groups, for one of which the name acute uræmia may well be retained, while the other might with more propriety be called chronic renal toxæmia.

*Cachexia.*—As a rule, as kidney disease advances the nutrition suffers greatly. Failure of health and strength in persons of middle age, without any evidence of malignant disease, should always excite the suspicion of granular kidney. This cachexia is characterised by anæmia and asthenia, and to some extent by loss of flesh, but emaciation is rarely carried to that degree which is met with in advanced malignant disease.

*Headache*, which is so common a symptom, often occurs in such severe paroxysm as to closely resemble migraine. It is usually vertical, but may be frontal or occipital.

*Renal Asthma.*—The dyspnœa, which may be paroxysmal, is very often cardiac, and usually due to bronchitis and emphysema. Any attack of the nature of true spasmodic asthma is very rare.

*Epileptiform Convulsions.*—Fits are, of course, the common form in which acute uræmia manifests itself, but epileptiform convulsions are not rare as one of the early symptoms of the late stage independent of uræmia. Curious attacks of cerebral irritation are not at all uncommon. They may take the form of attacks of general nervous irritability, of emotional excitement, or of almost maniacal delirium.

*Skin Affections in Renal Disease* fall into two groups according as there is general œdema or not. Where there is no œdema the rashes are probably of a toxic origin.

*Rashes without Œdema.*—Generally widespread, or even universal, they are of great obstinacy and of grave significance. They may be described as: (1) Erythema; (2) pityriasis rubra; (3) dermatitis exfoliativa; (4) general eczema; (5) a discrete, papular eruption, sometimeslichenous, sometimes resembling chronic urticaria. The association of a generalised skin eruption with albuminuria is of great importance, and justifies a more cautious prognosis than might otherwise be given.

Acute Uræmia.

The symptoms of acute uræmia have by no means that definite and uniform character which seems to be often assumed. Fits and coma are the two most characteristic symptoms, and yet patients may not have fits, or at any rate no marked convulsions, and they need not be comatose. In some cases the condition almost resembles that of narcotic poisoning. In others, again, symptoms of the most profound collapse develop, very much like those met with in acute ptomaine poisoning.

Whatever the form acute uræmia may take the prognosis is as grave as it can be. If any one of the forms has a less grave significance than the others, it is perhaps that in which there are epileptiform convulsions, for uræmic fits in granular kidney may end in recovery now and then, as they much more frequently do in acute nephritis. Frequently signs of cerebral irritation develop, the patient becomes extremely restless, sleepless, and more or less delirious, and sometimes passes into a condition of noisy, active delirium, not unlike delirium tremens, with which the condition may be confused. At other times the patient becomes violently maniacal, and is for the time a raging lunatic. The most interesting fact about uræmia in the course of granular kidney is that it may develop so suddenly, and with little or no warning, in the midst of apparent health, and carry the patient off.

Treatment.

Although we do not know at present how to prevent the disease developing and cannot restore the diseased organs to their normal condition, it does not follow that treatment is of no avail. The objects we should have in view in treatment are:—

1. To prevent the disease getting worse, if possible, and to relieve the damaged organ in every way possible. With this in view exposure must be avoided, as well as fatigue of either mind or body; the general health should be kept at its highest level; the diet should be appropriate, and, where feasible, the winter should be spent in a warm, dry, and genial climate.

2. To guard against the accidents specially likely to occur. These are failure of the heart and rupture of vessels. With this object violent exertion must be avoided, as well as all excessive mental work and anxiety; the patient should lead an easy life, both physical and mental; and, where unavoidable illness arises, such as a severe accident or acute pneumonia, if the weak spots be remembered much may be done to diminish the risks.

3. To counteract or relieve symptoms as they arise. It is often surprising when the cause upon which these symptoms depend has been recognised, that is to say, when granular kidney has been diagnosed, how much may be done to give relief.

Of drugs there are none more useful than nitrate of pilocarpin given in small doses two or three times a day by the mouth, or in urgent cases *sub cutem*. Thus the headache, irritability by day and restlessness by night, vomiting and digestive disturbance, the foul tongue and the dry skin, may all rapidly yield to a dose or two of pilocarpin, and the patient be restored to comfort, or even threatening symptoms of uræmia be removed.

There is one line of treatment which has not so far been investigated as fully as it deserves, and that is the treatment of chronic renal disease by means of renal extracts.

Renal extracts have hitherto not been used except in quite the latest stages of granular kidney at a time when probably the disease is too far advanced to be amenable to treatment at all. Yet, if we are to do any good at all by this or any other method, we must attack the disease before it has reached its later stages, and before the nutrition and general cachexia is so pronounced.

The use of renal extracts is still in a purely experimental stage. Judging by the analogy of myxœdema, it would be in the cases of chronic renal cachexia only that we should look for striking results, and this requires early and correct diagnosis.

